# Phenotypic characterization and candidate gene analysis of a short kernel and brassinosteroid insensitive mutant from hexaploid oat (*Avena sativa*)

**DOI:** 10.3389/fpls.2024.1358490

**Published:** 2024-04-26

**Authors:** Nikos Tsardakas Renhuldt, Johan Bentzer, Dag Ahrén, Sofia Marmon, Nick Sirijovski

**Affiliations:** ^1^ ScanOats Industrial Research Centre, Department of Chemistry, Division of Pure and Applied Biochemistry, Lund University, Lund, Sweden; ^2^ National Bioinformatics Infrastructure Sweden (NBIS), SciLifeLab, Department of Biology, Lund University, Lund, Sweden; ^3^ CropTailor AB, Department of Chemistry, Division of Pure and Applied Biochemistry, Lund University, Lund, Sweden

**Keywords:** seed shape, oats, *Avena sativa*, brassinosteroids, GSK3/SHAGGY-like kinase, mapping-by-sequencing, plant architecture, gene mapping

## Abstract

In an ethyl methanesulfonate oat (*Avena sativa*) mutant population we have found a mutant with striking differences to the wild-type (WT) cv. Belinda. We phenotyped the mutant and compared it to the WT. The mutant was crossed to the WT and mapping-by-sequencing was performed on a pool of F2 individuals sharing the mutant phenotype, and variants were called. The impacts of the variants on genes present in the reference genome annotation were estimated. The mutant allele frequency distribution was combined with expression data to identify which among the affected genes was likely to cause the observed phenotype. A brassinosteroid sensitivity assay was performed to validate one of the identified candidates. A literature search was performed to identify homologs of genes known to be involved in seed shape from other species. The mutant had short kernels, compact spikelets, altered plant architecture, and was found to be insensitive to brassinosteroids when compared to the WT. The segregation of WT and mutant phenotypes in the F2 population was indicative of a recessive mutation of a single locus. The causal mutation was found to be one of 123 single-nucleotide polymorphisms (SNPs) spanning the entire chromosome 3A, with further filtering narrowing this down to six candidate genes. In-depth analysis of these candidate genes and the brassinosteroid sensitivity assay suggest that a Pro303Leu substitution in AVESA.00010b.r2.3AG0419820.1 could be the causal mutation of the short kernel mutant phenotype. We identified 298 oat proteins belonging to orthogroups of previously published seed shape genes, with AVESA.00010b.r2.3AG0419820.1 being the only of these affected by a SNP in the mutant. The AVESA.00010b.r2.3AG0419820.1 candidate is functionally annotated as a GSK3/SHAGGY-like kinase with homologs in Arabidopsis, wheat, barley, rice, and maize, with several of these proteins having known mutants giving rise to brassinosteroid insensitivity and shorter seeds. The substitution in AVESA.00010b.r2.3AG0419820.1 affects a residue with a known gain-of function substitution in Arabidopsis BRASSINOSTEROID-INSENSITIVE2. We propose a gain-of-function mutation in *AVESA.00010b.r2.3AG0419820.1* as the most likely cause of the observed phenotype, and name the gene *AsGSK2.1*. The findings presented here provide potential targets for oat breeders, and a step on the way towards understanding brassinosteroid signaling, seed shape and nutrition in oats.

## 1 Introduction

Hexaploid oat (*Avena sativa*) is a staple cereal known for its high content of beta-glucan dietary fiber, protein, lipids, and other nutrients such as avenanthramides [EFSA Panel on Dietetic Products, Nutrition and Allergies (NDA) 2010; Zhang et al., 2021]. Nutrients are not uniformly distributed within oat kernels, and the proportion of nutrients differs across kernel tissues (Liu and Wise, 2021). It has previously been shown that seed shape and size are correlated to specialized metabolite abundance including beta-glucan content (Zimmer et al., 2021; Brzozowski et al., 2022). Disentangling the genetic determinants of seed size and shape could potentially help us understand connections to both oat nutrition and agricultural traits.

Hormones are involved in innumerable processes in plants, affecting plant growth and development in a multitude of ways. Mutations affecting plant hormone biosynthesis or signaling often result in phenotypes that alter many aspects of the plant, including plant size, architecture, placement of stomata, and a number of other properties, sometimes all at once (Smith et al., 2017). Grain size and shape have been extensively studied (Li et al., 2019) with several pathways shown to be involved in determining these phenotypes, many of them involving hormones. Seed shape is affected by both maternal and zygote genotypes. Phytohormone signaling plays a role in both maternal and zygote tissues. Brassinosteroids (BRs) are one class of plant hormones known to positively regulate seed size in several plant species (Tong et al., 2012; Jiang et al., 2013; Cheng et al., 2020; Kloc et al., 2020; Sun et al., 2021), promoting both cell expansion and cell proliferation in maternal tissues (Li et al., 2019). Auxins also affect seed shape through maternal tissues, with various auxin response factors having different effects on seed shape and size (Schruff et al., 2006; Hu et al., 2018; Jia et al., 2021). BRs affect seed size through zygotic tissues as well, regulating the HAIKU pathway together with abscisic acid, with cytokinins affecting seed size downstream of this pathway (Jiang et al., 2013; Li et al., 2013; Cheng et al., 2014). Auxins also affect seed size through zygotic tissues, with some controversy regarding which exact genes are involved in this (Bernardi et al., 2012; Ishimaru et al., 2013; Bernardi et al., 2019; Nonhebel et al., 2020; Kabir and Nonhebel, 2021). Beyond these, seed shape is influenced by a number of different mechanisms acting in maternal tissues, including G-protein signaling, transcriptional regulators, mitogen-activated protein kinase signaling, and through the ubiquitin-proteasome pathway (Li et al., 2019).

Extensive work has been performed in identifying genes affecting seed shape and size, with more than 80 genes and 400 quantitative trait loci (QTLs) identified only in rice (Li et al., 2022). Several of these are actively used in rice breeding programs (Zuo and Li, 2014). Mutants are commonly used to identify gene function. Mapping-by-sequencing is one way to map genes and does so by sequencing pools of recombinant plants sharing a phenotype of interest, similar to bulked segregant analysis (Schneeberger et al., 2009). For sufficiently large pools and with good enough phenotyping, recombination ensures that the mutant allele frequency will be close to one only in genomic regions that are close to the causative mutation, as the remaining mutations will have been lost randomly for the individuals within the pool. Mapping-by-sequencing has previously been employed to map the *AsCer-q* gene in oat (Kamal et al., 2022).

As a part of an oat TILLING population (Chawade et al., 2010), we have identified an oat (*Avena sativa*) mutant with short kernels, compact spikelets, and altered plant architecture when compared to the wild-type (WT) cultivar Belinda. In this paper, we characterize the mutant and provide evidence of BR insensitivity. We use a mapping-by-sequencing approach and the recently published oat cv. Sang reference genome (Kamal et al., 2022) to identify a likely causative gain-of-function mutation in a gene coding for a glycogen synthase kinase 3/SHAGGY-like kinase (GSK3/SHAGGY-like kinase, GSK), which we designate AsGSK2.1.

## 2 Materials and methods

### 2.1 Plant material and F2 segregating pools

The short kernel mutant was identified among the Belinda TILLING population (Chawade et al., 2010) during a field amplification experiment in 2017, and a forward genetics investigation of the genetic cause was initiated. Compared to the parental cv. Belinda, cv. Sang, and other members of the TILLING population, the mutant exhibited distinctly compact spikelets and short kernels and, to a lesser extent, altered plant architecture. The mutant phenotype was stable under greenhouse conditions with an 18 h photoperiod. A cross was made using Belinda as mother and the mutant as pollen donor (Belinda × mutant). The resulting F_1_ seeds were germinated in soil in a greenhouse and allowed to self-fertilize. Upon ocular inspection, the Belinda × mutant F_1_ plants resembled Belinda, indicative of the mutation being recessive. An F_2_ population was raised as in Kamal et al. (2022). Leaf tissue from 15 short kernel F_2_ plants was pooled for DNA extraction and sequencing. To study the mutant phenotype in more detail, eight non-backcrossed mutant plants and eight Belinda plants were grown in parallel in a greenhouse under ambient light conditions in Lund, Sweden, spring 2022.

### 2.2 Phenotyping

#### 2.2.1 Shoot and seed measurements

Photos of the growing plants were taken, and the height and the number of tillers at maturity were recorded. The primary tiller and two secondary tillers were individually collected for each plant, and the rest of the seeds were collected and threshed together using a Halderup LT-15 laboratory thresher. For a minimum of five plants, the primary panicle was characterized, and its total length, length of main branch of the first node (i.e., first whorl), and bottom panicle node distance were measured using a ruler. The length of the lower glume for the five top spikelets of the primary tiller was also measured with a ruler. The number of spikelets, the number of primary, secondary and tertiary seeds divided for each whorl, and the number of whorls were manually counted. Manually harvested seeds, divided into groups of primary, secondary, and tertiary seeds, were photographed and photos analyzed using the program SmartGrain (Tanabata et al., 2012) to determine seed length, seed width, area, perimeter length, and circularity.

#### 2.2.2 Macromolecular composition

The overall macromolecular composition was analyzed on threshed seeds, i.e., whole grains including hulls (n = 8) using near infrared spectroscopy with a handheld GrainSense calibrated for oat, giving an estimate of total lipids, protein, carbohydrates, and water content. Macromolecular composition was then measured using biochemical methods as described below for a selection of the samples. Around 40 of the seeds from each plant were manually dehulled and milled in a Fritsch Pulverisette 23 at 50 oscillations/s for 2 min. Milled oat was stored at −20°C until analysis.

#### 2.2.3 Protein content

Protein (n = 4) was measured using the Dumas method (Thermo Electron Corp., Flash EA, 1112 Series, Waltham, MA, USA, AOAC International, 2019, Method 990.03). For each sample, 25–27 mg was analyzed on a 33 mm tin disc. A tin disk without added sample was used for blank, aspartic acid was used as reference, and 5.4 (Mariotti et al., 2008) was used as nitrogen-to-protein conversion factor.

#### 2.2.4 Lipid content

Total lipids (n = 4) were extracted using chloroform:methanol at a 1:1 ratio according to a protocol adapted from Bligh and Dyer (1959). Shortly, the extraction used 3.75 mL of methanol/chloroform (2:1 v/v, with 0.05% butylated hydroxytoluene (BHT) w/v), 1 mL of 0.15 M acetic acid, 1.25 mL of chloroform, and 1.25 mL of water, with vortexing between each addition. An amount of 100 mg of milled seed material was used for extraction together with an internal standard of 0.40 mg of tripentadecanoin (Larodan). Extracted lipids were methylated according to Cavonius et al. (2014) and run on Thermo Scientific TRACE GC 1300-1310 Gas Chromatograph with a NUKOL™ fused silica capillary column (Supelco™). The GC program started at 80°C with 10°C increase per min to 150°C, 7°C increase per min until 215°C, and held at 215°C until a total runtime of 40 min. GLC426 (NuCheck) was used as an external reference standard.

#### 2.2.5 Beta-glucan content

Beta-glucan (n = 4) was analyzed using the McKleary method with the Megazyme kit (Megazyme, AOAC International, 2019, Method 995.16). The method was scaled down to work in 2 mL of sample tubes, using 2 × 15 mg (technical duplicates) of oat as starting material.

#### 2.2.6 Water content

Water content (n = 4) was measured gravimetrically on an analytical balance before and after freeze drying until stable weight.

#### 2.2.7 Brassinosteroid sensitivity

The sensitivity to BR was investigated with the leaf unrolling test (Wada et al., 1985; as described by Chono et al. (2003)). Samples were planted in vermiculite grown in darkness at 24°C for 8–9 days for mutant and WT plants. Leaf segments of 1.5 cm were incubated for 72 h in 10^−5^ M Epibrassinolide (Sigma-Aldrich) with water as control. The level of unrolling was measured using digital calipers. At least seven samples were used for the mutant and WT, respectively, and the experiment was repeated three times.

#### 2.2.8 Statistics

Phenotype statistics were calculated in R (R Core Team, 2023, v4.2.3), with Wilcoxon rank sum tests used to compute p-values, and values of p < 0.05 were considered significant. The tidyverse packages (Wickham et al., 2019) and gtsummary (Sjoberg et al., 2021) were used in data processing and table creation.

### 2.3 DNA extraction, genome resequencing, and variant calling and analysis

Whole-genome short-read resequencing of the pooled tissue was done as that in the work of Kamal et al. (2022). The mutant tissue pool was ground to a fine powder using liquid nitrogen in a mortar and pestle. Genomic DNA was isolated using the Qiagen DNeasy Plant Mini Kit according to the manufacturer’s protocol. A library of 350-bp insert size was prepared using the Illumina TruSeq PCR-free library preparation kit (Illumina, San Diego, CA). Samples were sequenced on NovaSeq6000 (NovaSeq Control Software 1.6.0/RTA v3.4.4) with a 2 × 151 setup using “NovaSeqXp” workflow in “S1” or “SP” mode flowcell to generate reads corresponding to 30× coverage. The Bcl to FastQ conversion was performed using bcl2fastq_v2.20.0.422 from the CASAVA software suite. The quality scale used is Sanger/phred33/Illumina 1.8+. The reads were trimmed using fastp (Chen et al., 2018; v0.20.1, options - -correction - -adapter_sequence=AGATCGGAAGAGCACACGTCTGAACTCCAGTCA- -adapter_sequence_r2=AGATCGGAAGAGCGTCGTGTAGGGAAAGAGTGT), mapped to the *Avena sativa* cv. Sang reference genome (Kamal et al., 2022) using BWA-MEM2 (Vasimuddin et al., 2019; v2.2.1), and variants were called using DeepVariant (Poplin et al., 2018; v1.1.0), GNU Parallel (Tange, 2021; v20210422), and GLNexus (Lin et al., 2018; Yun et al., 2021; v1.3.1). The full variant calling pipeline is available at GitHub at nikostr/dna-seq-deepvariant-glnexus-variant-calling (Tsardakas Renhuldt, 2021; v0.3.1) and was run using Snakemake (Mölder et al., 2021; v6.5.1). Variants in independent lines from the TILLING population as well as in Sang or Belinda (**Supplementary Table S1**) were called as a part of Kamal et al. (2022) and filtered out using bcftools (Danecek et al., 2021, v1.12). SnpEff (Cingolani et al., 2012, v4.3.1t) and v1.1 of the Sang annotation of protein coding genes (Kamal et al., 2022) were used to annotate effects of single-nucleotide polymorphisms for the variants.

The mutant allele frequency of variants with a total allelic depth ≥ 15 was plotted and used to identify conserved regions among the pooled samples. Variants were filtered on the basis of read support (read depth of alternate allele ≥ 15, no reads supporting the reference allele), being located in a region found to be conserved in the pool, being classified by SnpEff as having moderate or high impact, and being located in a gene with expression (>0.5 transcripts per million) in all seed, glume, and spikelet tissue samples. Gene expression values in transcripts per million (three developing seed samples from cv. Sang, seven glume samples, and eight spikelet samples from cv. Belinda, and 21 additional developing seed samples) as provided by Kamal et al. (2022) were used (**Supplementary Table S2**). The filtered genes were investigated individually. Orthogroups from Kamal et al. (2022) were used to identify genes homologous to the genes affected in the mutant. Multiple sequence alignments were produced using MUSCLE (Edgar, 2004, v3.8.1551), with fasttree (Price et al., 2010; v2.1.10) used to build phylogenetic trees, which, in turn, were visualized using iTOL (Letunic and Bork, 2021; v6.7).

Oat genes potentially involved in the regulation of seed shape were also identified using the orthogroups and a recent review of such genes (Li et al., 2019). Only genes from species included in the orthogroups (*Arabidopsis*, rice, wheat, and maize) were considered. The Sang positions of the markers used by Zimmer et al. (2021) were taken from Tinker et al. (2022). We used KofamScan (Aramaki et al., 2020), v1.3.0, to search the Sang proteins against the eukaryote-specific profiles in the Kofam database (https://www.genome.jp/ftp/db/kofam/archives/2021-12-01/). Only KEGG Orthology matches with scores above the pre-computed KofamScan thresholds were considered.

## 3 Results

**Figure 1:**
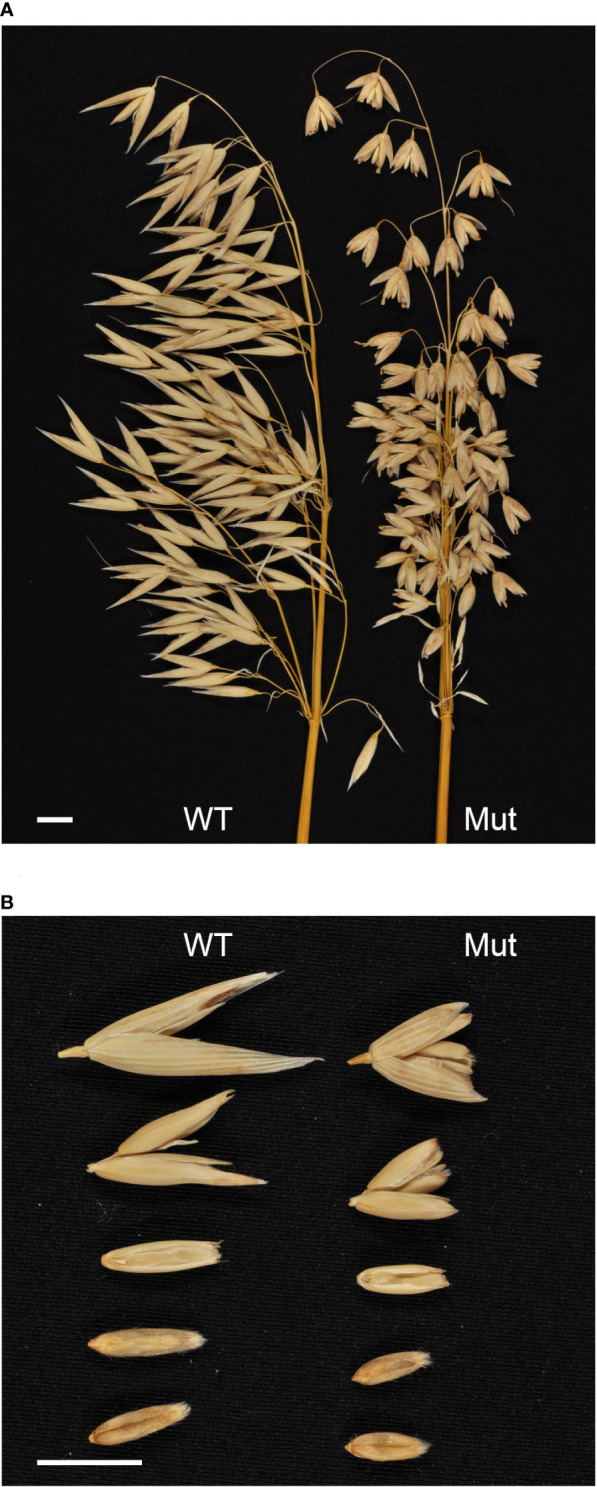
Comparison of cv. Belinda (WT) and mutant (Mut) phenotypes. White bars correspond to 1 cm. **(A)** Comparison of panicles, showing altered panicle architecture in the mutant. **(B)** Comparison of spikelets and kernels, showing smaller spikelets and kernels in the mutant.

The oat mutant was initially identified in a TILLING population (Chawade et al., 2010) due to its strikingly different panicles. Out of the 69 F_2_ plants, 15 shared the short kernel phenotype. This ratio is in line with what would be expected of a recessive trait caused by a single locus (null hypothesis is that homozygous mutant allele:heterozygous + homozygous WT allele have distribution 1:3, x^2^(1, N = 69) = 0.39, P = 0.53). **Figure 1** shows a comparison of the mutant to cv. Belinda. The mutant kernels and glumes were significantly (Wilcoxon rank sum test, p ≤ 0.03) shorter than the corresponding tissues in the mother variety Belinda; see **Table 1**. Only the length of the seeds and associated tissues were affected, no change was seen in width, nor were the seeds misshaped, as shown in **Figure 1B**. The shorter seeds also had a significantly lower seed area (mutant, 18.9 ± 1.3 mm^2^; cv. Belinda, 28 ± 0.5 mm^2^) and thousand kernel weight (mutant, 27 ± 2.2 g; cv. Belinda, 39 ± 2.6 g). Studying the plant phenotype, it was evident that many different parts of the plant were affected and that the effects were not limited to the seeds and associated tissues being short. Total plant height, both at intermediate growth (observed, not quantified) and at maturity, as well as distance between bottom panicle nodes and length of the main branch of the first node were significantly shorter than in the control plants. Total panicle length in the mutant was not shorter than the control, despite the shorter node distances. This seems to be a result of altered panicle architecture, with, in general, one additional whorl. The higher whorls (three and up) also contained a significantly higher number of seeds in the short kernel panicles. Further, the mutant spikelets also more often contained tertiary grains. The nutritional quality of the short kernel oat was also somewhat altered, with the protein content being higher than in the mother cultivar (mutant, 19.3 ± 1.3%; cv. Belinda, 16.7 ± 1.0%, according to the Dumas method described above), and a corresponding lower carbohydrate content (**Supplementary Table S3**). The beta-glucan content and lipid content were similar in both seed types.

**Table 1:** Characteristics of the plants and the seeds of cv. Belinda and the mutant.

	Belinda		Mutant		
Characteristic	N^1^	Mean (SD^2^)	N^1^	Mean (SD^2^)	p-value^4^
Plant height (cm)	8	118.9 (6.9)	8	103.1 (2.9)	<0.001
Main panicle length (mm)	6	212.5 (10.3)	6	197.7 (14.2)	0.054
Main branch of first node (mm)	6	118.8 (13.2)	6	93.3 (1.6)	0.005
Bottom panicle node distance (mm)	6	59.0 (3.3)	6	48.0 (3.2)	0.005
Lower glume length (mm)	6	21.6 (0.7)	6	10.8 (0.3)	0.005
Kernel length including hull (mm)^3^	4	13.6 (0.3)	4	8.6 (0.4)	0.030
Kernel width including hull (mm)^3^	4	2.8 (0.1)	4	2.9 (0.1)	0.4
Kernel area (sq. mm)^3^	4	28.0 (0.5)	4	18.9 (1.3)	0.030
Number of kernels	6	140.2 (22.2)	6	179.0 (29.7)	0.045
Number of tertiary kernels	6	1.5 (2.1)	6	16.5 (7.9)	0.005
TKW for primary and secondary kernels (g)	6	39.0 (2.6)	6	26.7 (2.2)	0.005

^1^Number of individuals used to calculate mean and standard deviation (SD).

^2^Standard deviation.

^3^P-values are based on Wilcoxon rank sum test comparisons of Belinda and the mutant.

^4^Based on averages of >98 seeds per replicate.

**Figure 2:**
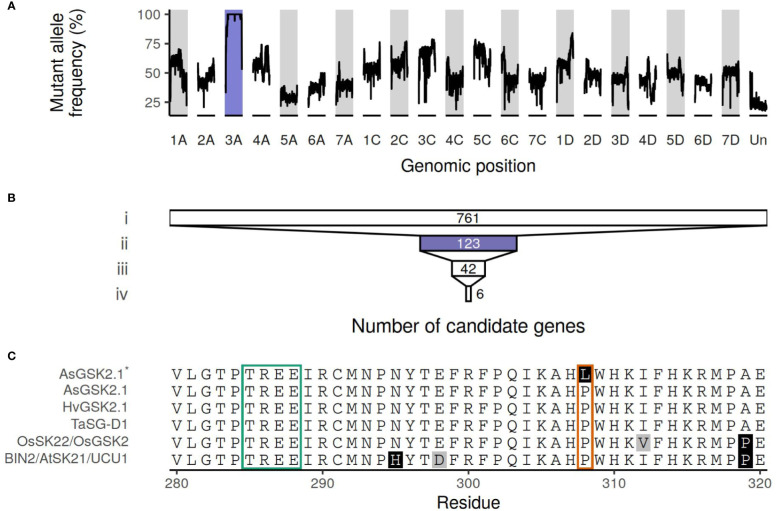
**(A)** Median allele frequency computed as a sliding window over the called variants. A window of 100 variants (total allelic depth *≥* 15) was used. **(B)** The number of genes remaining after filtering steps. i) Genes with a mutation that is classified as having high or moderate impact by SnpEff; ii) that are also located on chromosome 3A; iii) and have *≥* 15 reads supporting the alternate allele and no reads supporting the reference allele (**Supplementary Figure S2**); iv) and that are also expressed in all seed, spikelet, and glume samples (**Supplementary Figure S1**). Purple highlights chromosome 3A. **(C)** Truncated multiple sequence alignment of AsGSK2.1, the sequence of the mutant AsGSK2.1^*^, BIN2/AtSK21/UCU1, HvGSK2.1, OsSK22/OsGSK2, and TaSG-D1. Amino acids with white background are conserved in at least half of the sequences, in gray amino acids similar to the majority amino acid, other amino acids indicated in black. The boxes correspond to positions where known gain-of-function substitutions have been identified previously. The green box highlights the highly conserved TREE motif known to contain multiple gain-of-function substitutions in *Triticum sphaerococcum* and *Arabidopsis*. The orange box corresponds to the residue affected by the weaker *ucu1-3* gain-of-function mutation in *Arabidopsis* and is also the location of the Pro303Leu substitution in AsGSK2.1, here shown in AsGSK2.1^*^. The full protein sequence alignment is shown in **Supplementary Figure S3**.

The mutant allele frequency was higher across chromosome 3A than throughout the remainder of the genome, as shown in **Figure 2A**. The number of genes remaining when performing filtering as outlined above is shown in **Figure 2B**, with a total of six genes remaining following all filtering steps: characteristics and orthology information regarding these candidate genes are shown in **Tables 2** and **3**, respectively. **Supplementary Table S4** connects previously published gene names to the gene identifiers present in the orthogroups. All six candidate genes had other expressed oat genes present in the same orthogroup (**Supplementary Figure S1**). Out of the six genes, two had orthologs known to have some effect on seed shape.

**Table 2:** Functional annotation, KEGG orthology, and mutation of the proteins corresponding to the six candidate genes.

Candidate	Functional annotation	KEGG orthology	Mutation
AVESA.00010b.r2.3AG0417810.1	NAD(P)-binding Rossmann-fold superfamily protein	K13606 (chlorophyll(ide) b reductase [EC:1.1.1.294])	Gly122Glu
AVESA.00010b.r2.3AG0419820.1	SHAGGY-like kinase	K14502 (protein brassinosteroid insensitive 2 [EC:2.7.11.1])	Pro303Leu
AVESA.00010b.r2.3AG0424310.1	RNA exonuclease 4	K18327 (R exonuclease 4 [EC:3.1.-.-])	Gly273Asp
AVESA.00010b.r2.3AG0426560.1	Hyp O-arabinosyltransferase-like protein	K20782 (hydroxyproline O- arabinosyltransferase [EC:2.4.2.58])	Ala76Val
AVESA.00010b.r2.3AG0430410.1	DCD (Development and Cell Death) domain protein		Pro290Ser
AVESA.00010b.r2.3AG0437710.1	Transcriptional corepressor LEUNIG		Gly558Asp

**Table 3:** Notable published homologs of the candidate genes.

Candidate	Description	Homolog	Reference
*AVESA.00010b.r2.3AG0417810.1*			
	NYC1 encodes a chlorophyll b reductase. Deleterious mutations in this gene leads to hindered chlorophyll degradation/yellowing during sencescense.	AtNYC1 OsNYC1	Horie et al. (2009) Kusaba et al. (2007)
*AVESA.00010b.r2.3AG0419820.1*			
	BIN2 is known to be involved in BR signaling. Mutations affecting the TREE motif are known to cause gain-of-function, repressing BR signaling. In barley and rice knockouts of homologous genes lead to increased grain size. Gain-of-function mutation in the wheat ortholog and overexpression of rice ortholog OsSK22 leads to smaller, rounder kernels and shorter stature.	BIN2/AtSK21	Pérez-Pérez et al. (2002); Peng and Li (2003)
		HvGSKs	Kloc et al. (2020)
		OsSK22, OsSK23, OsSK41	Tong et al. (2012); Hu et al. (2018); Gao et al. (2019)
		TaSG-D1	Cheng et al. (2020)
*AVESA.00010b.r2.3AG0424310.1*			
	The homologue in Arabidopsis contains exonuclease domain, but has no documented effect on miRNA.	AT3G15080.1	Ramachandran and Chen (2008)
*AVESA.00010b.r2.3AG0426560.1*			
	MtRDN1 is involved in root nodule formation in Medicago truncatula. OsRDN1 is capable of rescuing MtRDN1 mutants. HPAT3 is involved in pollen tube formation in Arabidopsis.	MtRDN1	Schnabel et al. (2011); Kassaw et al. (2017)
		OsRDN1	Schnabel et al. (2011); Kassaw et al. (2017)
		HPAT3	Beuder et al. (2020)
*AVESA.00010b.r2.3AG0430410.1*			
	Involved in *Arabidopsis* stress response and inhibits cell death.	NRP	Yang et al. (2021)
*AVESA.00010b.r2.3AG0437710.1*			
	Transcriptional corepressor involved in floral organ identity. Mutants are known to have reduced fertility, and may produce abnormal seeds.	LEUNIG OsLUGL	Liu and Meyerowitz (1995) Yang et al. (2019)

The mutant had a C to T substitution at chr3A:64189534 (32 reads supporting the alternate allele, no reads supporting the reference allele, QUAL value of 61.7) affecting *AVESA.00010b.r2.3AG0419820.1* (**Supplementary F** **igure S2**). This leads to a Pro303Leu substitution in the protein, which is annotated as a SHAGGY-like kinase. The protein belongs to the same orthogroup (OG0000448) as *Arabidopsis* BIN2 (also known as AtSK21 and UCU1), as well as the barley HvGSK proteins, and OsSK/OsGSK from rice. It is also homologous to TaSG-D1 in wheat. **Figure 2C**; **Supplementary Figure S3** show multiple sequence alignments of homologous proteins previously known from the literature, including positions of known gain-of-function substitutions from *Arabidopsis* and wheat. **Supplementary Figure S4** shows a phylogenetic tree of OG0000448 and TaSG-D1 with subgroups indicated along with proteins previously known from other species (Cheng et al., 2020; Kloc et al., 2020; Li et al., 2021; Hou et al., 2022; Li et al., 2022; Zhang et al., 2022). The Pro303Leu substitution affects the same residue as the weak gain-of-function mutation *ucu1-3* in *Arabidopsis*. These homologous proteins are known negative regulators of BR signaling, with mutants known to affect kernel size and plant architecture (Pérez-Pérez et al., 2002; Tong et al., 2012; Cheng et al., 2020; Kloc et al., 2020). The second candidate protein with orthologs known to affect seed shape is encoded by *AVESA.00010b.r2.3AG0437710.1*, orthologous to the transcriptional corepressor LEUNIG and OsLUGL. When *OsLUGL* is mutated in rice, it results in seeds that are deformed and reduced seed set, neither of which were observed in the short kernel mutant. Mutation in *OsLUGL* also does not affect plant height and overall architecture (Yang et al., 2019), whereas the contrary was observed in the mutant studied here. Further, we had identified a mutation in *AVESA.00010b.r2.3AG0437710.1* in another oat line lacking the phenotype (data not shown). Thus, the Gly558Asp substitution in AVESA.00010b.r2.3AG0437710.1 is unlikely to be the cause of the mutant phenotype. A functional orthology analysis of the remaining four candidate genes (*AVESA.00010b.r2.3AG0417810.1*, *AVESA.00010b.r2.3AG0424310.1*, *AVESA.00010b.r2.3AG0426560.1*, and *AVESA.00010b.r2.3AG0430410.1*) revealed potential biological functions that include a chlorophyll reductase, exonuclease, hydroxyproline O-arabinosyltransferase, and a DCD domain (Development and Cell Death)–containing protein, respectively. A detailed investigation of the literature on orthologs of these proteins did not provide support for these candidates playing a role in seed shape or plant architecture, and we therefore considered the identified mutations in these genes to be very unlikely to be responsible for the mutant phenotype.

To further study if the identified amino acid substitution in the SHAGGY-like kinase candidate AVESA.00010b.r2.3AG0419820.1 affects BR signaling in the oat mutant, a leaf unrolling test was performed (Wada et al., 1985; as described by Chono et al. (2003)). The Belinda control plants were significantly more sensitive to BR as shown by the test, with the leaves being 90(±14)% unrolled compared to the mutant leaves being only 58(±11)% unrolled (Wilcoxon rank sum test, p = 0.004) with no difference (Wilcoxon rank sum test, p = 0.13) between the samples subjected to water, 36(± 6)% compared to 39(± 2)% unrolling for the Belinda control and the mutant, respectively.

To help exclude alternate genes causing the phenotype, gene candidates were identified on the basis of homology to previously published seed shape genes from other species. Out of the 89 *Arabidopsis*, wheat, rice, and maize proteins listed in the review of regulators of seed size control written by Li et al. (2019), an orthogroup was identified for 86 of these, corresponding to a total of 63 orthogroups containing a total of 298 oat proteins, five of which are encoded by genes located on chromosome 3A. Among all oat genes identified through these orthogroups, only the SHAGGY-like kinase *AVESA.00010b.r2.3AG0419820.1* contains a mutation classified as having at least moderate impact by SnpEff. The full list of candidate seed shape proteins can be found in **Supplementary Table S5**, and the locations of their corresponding genes are shown together with the seed shape markers from Zimmer et al. (2021) in **Supplementary F** **igure S5**.

Taking the above into account, we propose the Pro303Leu substitution in AVESA.00010b.r2.3AG0419820.1 is a gain-of-function mutation that is most likely among the six candidates to cause the oat short kernel phenotype reported herein. Following accepted naming convention, we designate AVESA.00010b.r2.3AG0419820.1 as AsGSK2.1.

## 4 Discussion

Above, we have identified a substitution in the protein encoded by *AVESA.00010b.r2.3AG0419820.1* as the likely cause of the mutant phenotype. Beyond the experimental evidence presented here, it is the only homolog of seed shape proteins identified in Li et al. (2019) that is found to be affected in the mutant. The candidate is homologous to GSK3 proteins, including *Arabidopsis* BIN2, as well as TaSG-D1 in wheat, the HvGSK proteins in barley, and OsSK/OsGSKs in rice. In *Arabidopsis*, the GSK3/SHAGGY-like kinase BIN2 has been extensively characterized as a transcriptional corepressor of BR signaling, with several known gain-of-function mutations that give a BR-deficient phenotype, including dwarfing (Pérez-Pérez et al., 2002). It has also been shown to interact with and phosphorylate auxin response factors in *Arabidopsis*, with its homolog OsSK41/OsGSK5 similarly phosphorylating OsARF4 in rice (Cho et al., 2014; Hu et al., 2018). In wheat, barley and rice, there is evidence that these affect kernel shape and size, with the gain-of-function mutation affecting TaSG-D1 being known to produce small, round seeds as well as altered plant architecture in *Triticum sphaerococcum* (Cheng et al., 2020). The Pro303Leu substitution in the protein encoded by the candidate gene causing a gain-of-function, similar to the *Arabidopsis ucu1-3* mutation (Pérez-Pérez et al., 2002), would help explain how this single mutation causes the mutant phenotype in spite of there being a total of 25 oat proteins with evidence of RNA expression present in the same orthogroup. That the phenotype of the mutant studied here diverges from the WT in many different parts of the plant is in line with what is seen for other mutants of GSK3 proteins (see, e.g., Song et al. (2023) and Li et al. (2021)). That the causative mutation affects hormone signaling is also expected given the systemic effect seen in the mutant.

Youn and Kim (2015) proposed to unify the naming of the GSK3/SHAGGY-like kinases in *Arabidopsis* and rice, with the first digit of the gene name given by the subgroup (I–IV). This naming scheme has not been universally adopted. In this paper, we opt for a naming scheme similar to that used in barley, where the digit prior to the dot indicates the subgroup. Subgroups are indicated in **Supplementary Figure S** **4**.

For growers of oat, Yan et al. (2013) identify several ideal traits for a milling oat cultivar: high and stable grain yield, resistance to lodging, proper maturity, resistance to relevant biotic and abiotic stresses, high test weight, large kernels, and high straw yield. Among the ideal traits relevant for consumers, they include a high beta-glucan content. The identification of *AsGSK2.1* and its homologs provides a potential target for oat breeders. Downregulating the *HvGSK* genes has previously been shown to give barley with higher thousand kernel weight under normal growing conditions, as well as increased biomass in both normal growing conditions and under salt stress (Kloc et al., 2020). In rice, upregulation of *OsSK22*/*OsGSK2* gives rise to short, round grains, and suppression of the same gene gives longer grains (Tong et al., 2012). Loss-of-function mutations affecting *OsSK41*/*OsGSK5* or *OsSK23*/*OsGSK3* cause larger and heavier grain (Hu et al., 2018; Gao et al., 2019). Downregulation or knock-outs of *AsGSK2.1* could potentially provide oats with larger kernels and increased oat yields. Achieving such downregulation might seem daunting given the large number of oat genes in the orthogroup, but it has been shown that expression is positively correlated among the paralogs in barley (Kloc et al., 2020), meaning that it may not be necessary to target all 25 oat genes of the orthogroup to see effects on grain shape and weight.

Beyond targeting the identified candidate gene and its homologs directly, these findings may function as a stepping stone in helping to understand BR signaling in oats and monocots more widely. BR signaling in cereals and monocots, in general, is not as well understood as that in *Arabidopsis*, in spite of BR playing an important role affecting yield and yield-related agronomic traits. Of particular note are semi-dwarf cultivars with lower risk of lodging and more erect cultivars allowing for increased planting density, which, in turn, contributes to a higher yield. BR affects both of these phenotypes (Gruszka, 2020). Even though the GSKs are a conserved part of BR signaling across multiple species including cereals, further studies of this mutant and the candidate gene could provide insight into other parts of oat BR signaling, which may be of relevance in other cereals as well.

In the populations studied by Zimmer et al. (2021), they identified negative correlations between kernel width/thickness and beta-glucan content, but no real correlation between kernel length and beta-glucan content. Our measurements show no significant difference neither in the kernel width nor in the beta-glucan content between the mutant and cv. Belinda. If there is no correlation between beta-glucan content and kernel length and if suppressing *AsGSK* genes does indeed produce longer seeds similar to the effect of suppressing *OsGSK2*/*OsSK22* (Tong et al., 2012), then suppressing the *AsGSK* genes may potentially produce larger seeds without negatively affecting the beta-glucan content.

QTLs for kernel length, width, and thickness were also identified by Zimmer et al. (2021), with length QTLs being located in Mrg06, Mrg21, and Mrg24, and QTLs for width and thickness located in Mrg13. Using the recently published reference genomes and their corresponding nomenclature (International Oat Nomenclature Committee, 2021), these merge groups correspond to chromosomes 5D, 4D, 5A, and 2C, respectively. Out of the genes corresponding to the proteins present in OG0000448, one is located on 2C and two are located on 4D, but, on both chromosomes, there are other potential candidates located closer to the significant markers. None of the significant markers from Mrg24 are present on chromosome 5A in the Sang assembly. On chromosome 2C, we note that a gene coding for a protein in the same orthogroup as DA1, DAR1, and ZmDA1 (OG0008367, AVESA.00010b.r2.2CG0324970) is expressed in the relevant tissues and is located in the middle of the region containing significant markers. A member of the same orthogroup as the known grain width related protein GW8 (OG0002672, AVESA.00010b.r2.2CG0325500) is located very close to these markers as well, but it is not present in the expression data. We also note that genes with homologs known to affect grain length are located close to the grain length QTLs on chromosomes 4D and 5D (AVESA.00010b.r2.4DG0789350 in orthogroup OG0000353 with TGW6; AVESA.00010b.r2.5DG0996410 in OG0017199 with GS3) but that these are not reliably expressed in the expression data that we consider here. The above is one demonstration of how the list of potential grain shape genes presented in **Supplementary Table S5** may be used, and we hope that it will prove to be a useful resource for breeders as well as for future studies of kernel shape in oats.

We have identified a likely gain-of-function mutation in *AsGSK2.1* (*AVESA.00010b.r2.3AG0419820.1*), as the most likely candidate mutation causing the mutant to display a short stature, compact panicles, short kernels, and an altered plant architecture. We have also identified oat proteins homologous to proteins previously known to affect kernel shape in other plant species. These findings provide potential targets for oat breeders, and a step on the way toward understanding BR signaling, seed shape, and nutrition in oats.

## Data availability statement

The raw whole genome resequencing data have been deposited in the European Nucleotide Archive at EMBL-EBI under accession number PRJEB57056 https://www.ebi.ac.uk/ena/browser/view/PRJEB57056. The KEGG Orthology is available at doi: 10.5281/zenodo.7886703.

## Author contributions

NT: Conceptualization, Data curation, Formal analysis, Investigation, Methodology, Project administration, Software, Validation, Visualization, Writing – original draft, Writing – review & editing. JB: Conceptualization, Data curation, Formal analysis, Investigation, Methodology, Software, Validation, Visualization, Writing – original draft, Writing – review & editing. DA: Conceptualization, Methodology, Supervision, Writing – original draft, Writing – review & editing. SM: Conceptualization, Data curation, Formal analysis, Investigation, Methodology, Project administration, Resources, Supervision, Validation, Writing – original draft, Writing – review & editing. NS: Conceptualization, Data curation, Formal analysis, Investigation, Methodology, Project administration, Resources, Supervision, Validation, Writing – original draft, Writing – review & editing.
