# Supplementary material for: Phenotypic characterization and candidate gene analysis of a short kernel and brassinosteroid insensitive mutant from hexaploid oat (Avena sativa)
Source: Front Plant Sci. 2024 Apr 26;15:1358490. doi: 10.3389/fpls.2024.1358490 (PMC11082396; doi:10.3389/fpls.2024.1358490)
Supplement: Supplementary file 1 [file DataSheet_1.zip › Supplementary-figures-S1-S4.pdf]

Supplementary Material

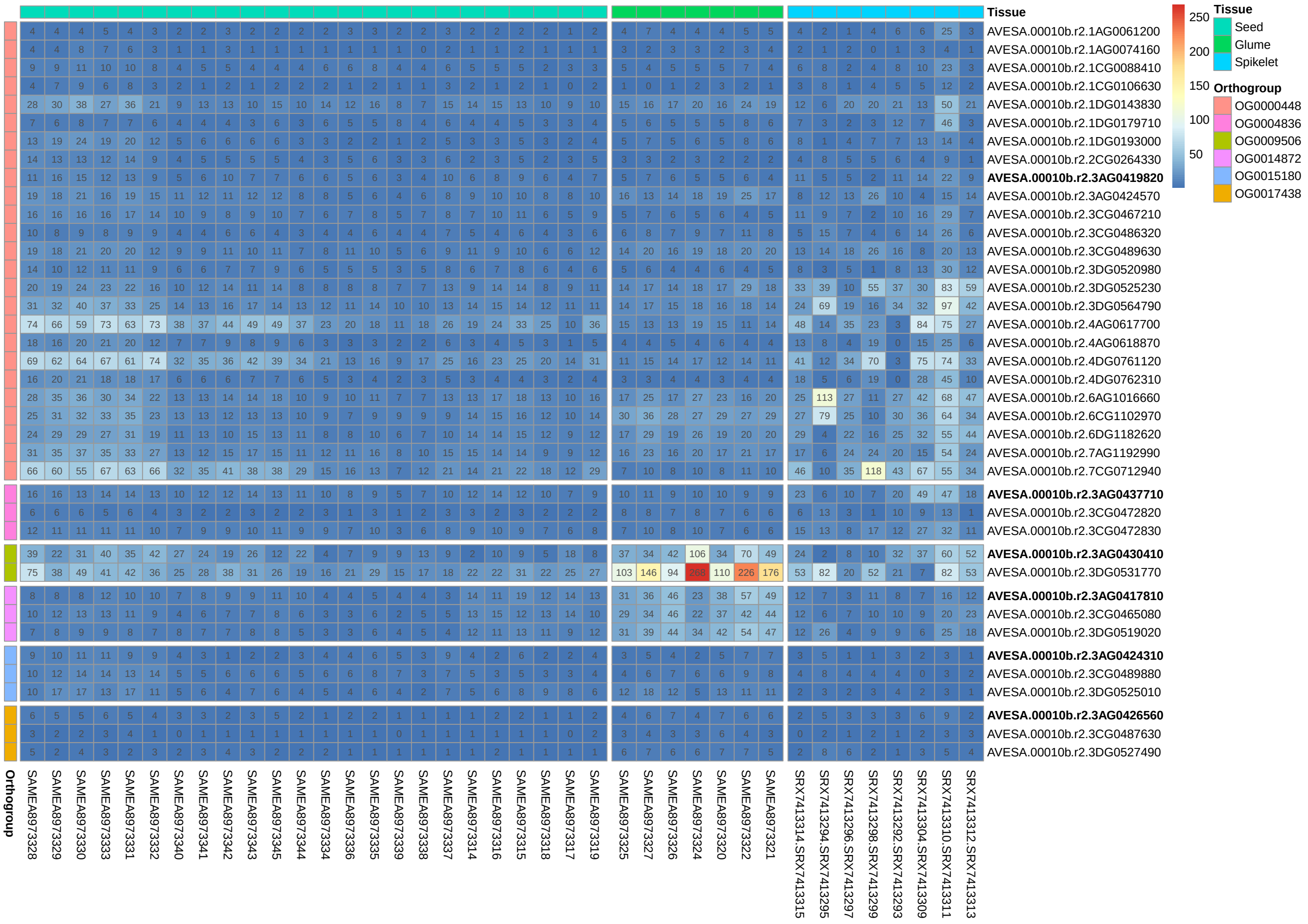

Figure S1. Expression values (transcripts per million) per RNA-seq sample of all genes sharing an orthogroup with the candidate genes. Candidate gene IDs are bolded. RNA-seq sample names correspond to ENA (European Nucleotide Archive) sample or experiment accessions.

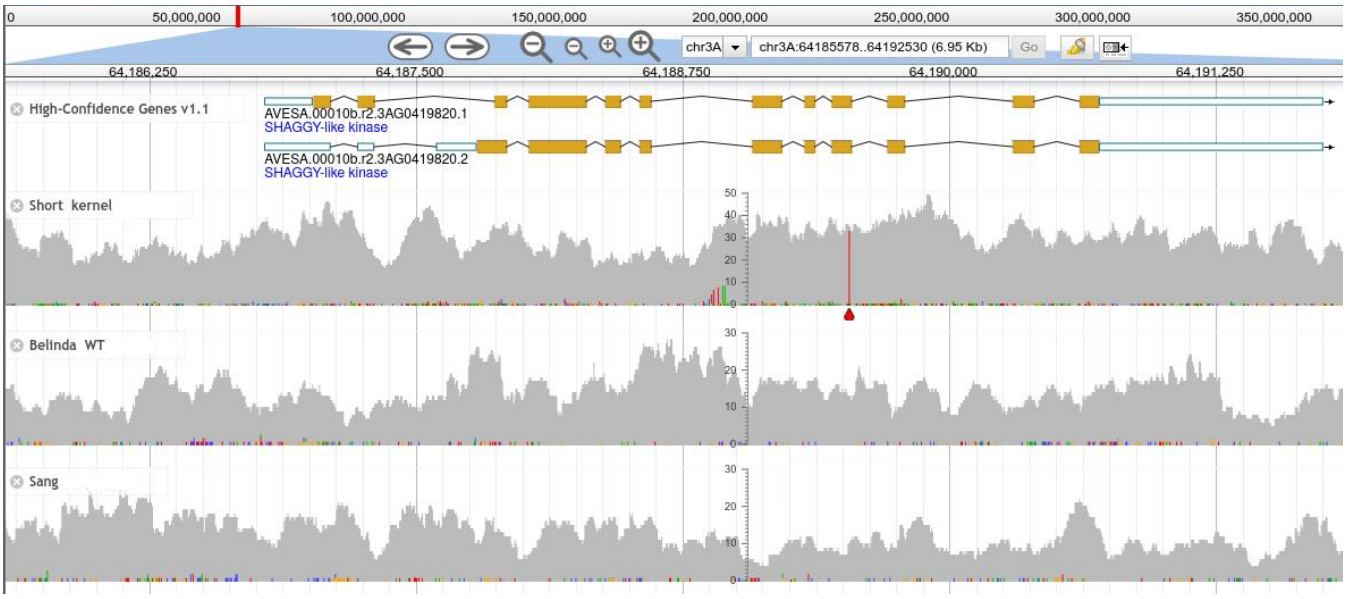

Figure S2. Sequencing depth and SNP discovery in the candidate gene *AVESA.00010b.r2.3AG0419820.1*. The candidate gene and the mapped reads (grey shading) for the pooled mutant short kernel samples, as well as Belinda WT (ENA sample accession: SAMEA8973507) and a resequenced sample from the reference cultivar Sang (ENA sample accession: SAMEA8973508) originally sequenced as a part of Kamal et al. (2022). Colored bars in the coverage graph indicate frequency of bases not matching the reference sequence. The vertical red line indicates that all the reads mapping in this position share the same substitution (C instead of T), and this is what is being called as a variant/mutation. The vertical scales in the center of each sample row indicate the short-read coverage.

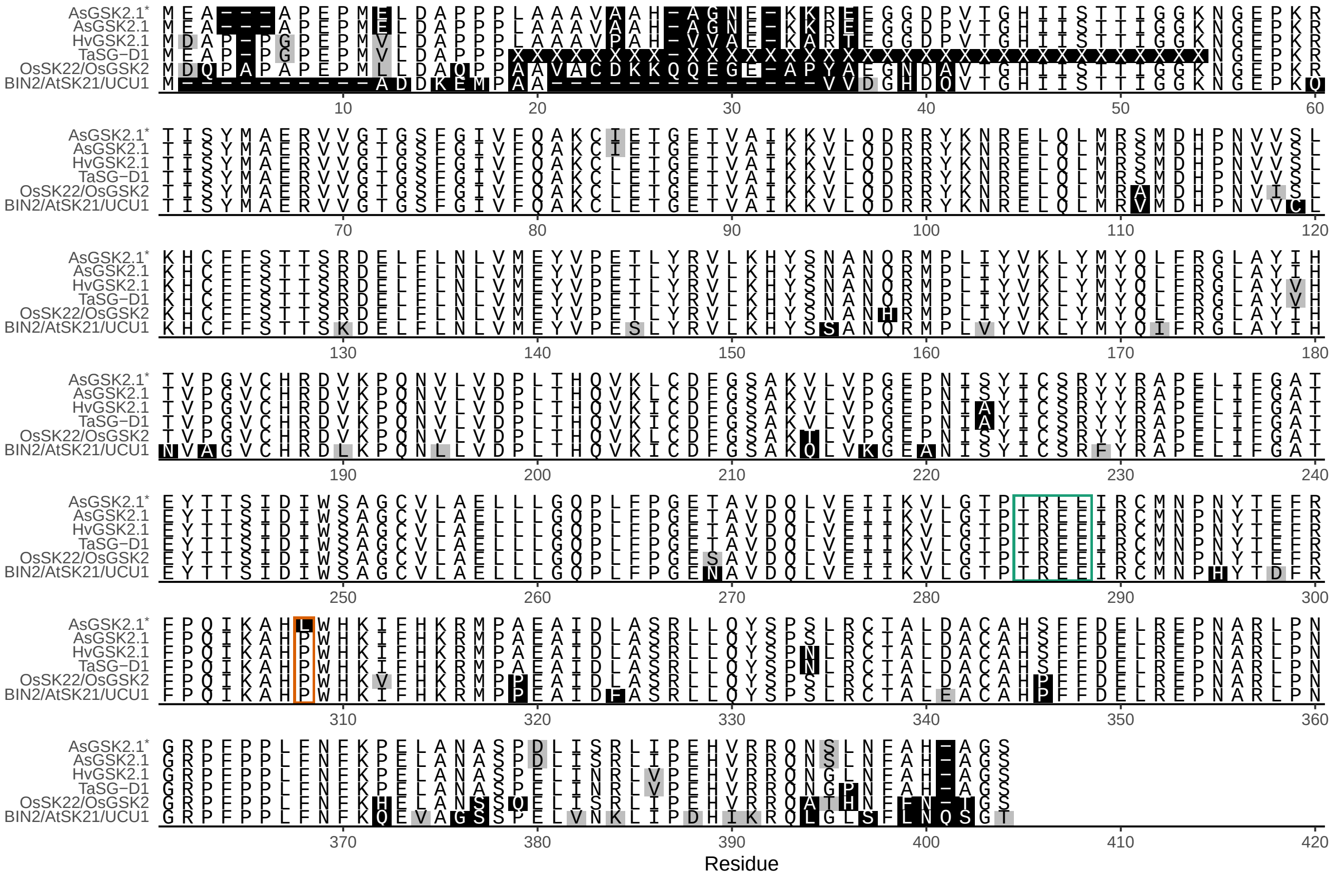

Figure S3. Multiple sequence alignment of AsGSK2.1, the sequence of the mutant AsGSK2.1*, BIN2/AtSK21/UCU1, HvGSK2.1, OsSK22/OsGSK2 and TaSG-D1. Amino acids with white background are conserved in at least half of the sequences, in gray amino acids similar to the majority amino acid, other amino acids indicated in black. The boxes correspond to positions where known gain-of-function substitutions have been identified previously. The green box highlights the highly conserved TREE motif known to contain multiple gain-of-function substitutions in *Triticum sphaerococcum* and *Arabidopsis*. The orange box corresponds to the residue affected by the weaker *ucu1-3* gain-of-function mutation in *Arabidopsis*, and is also the location of the Pro303Leu substitution in AsGSK2.1, here shown in AsGSK2.1*.

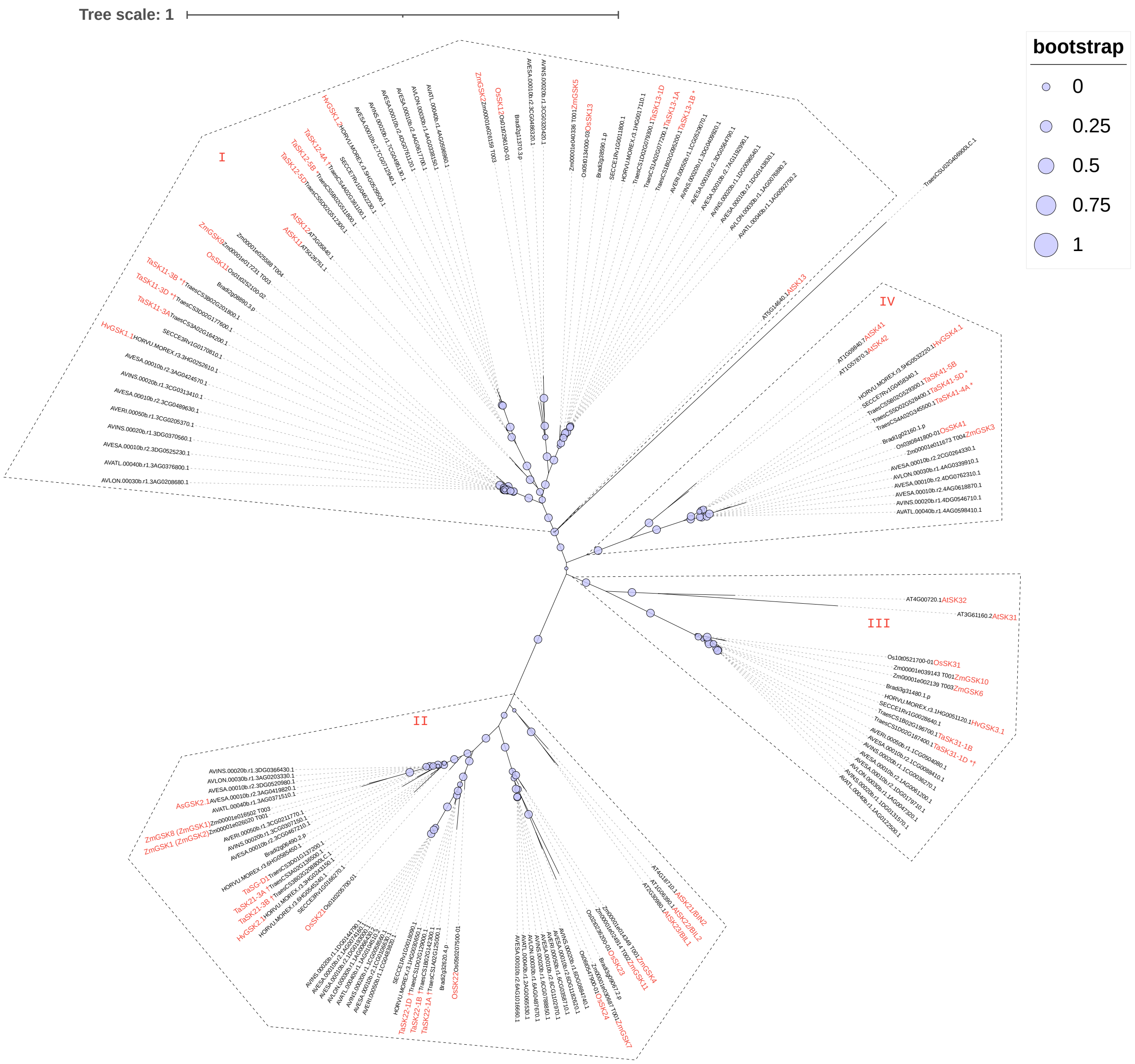

Figure S4. Phylogenetic tree of orthogroup OG0000448 and TaSG-D1. GSK3 family subgroups are indicated by dotted lines and named (I-IV). Protein names are shown in red. Bootstrap values correspond to fasttree local support values. Cheng et al. (2020) designate TraesCS3D01G137200 as TaSG-D1. TraesCS3D02G137200.1 is the best but not perfect match for TaSK21-3D (Zhang et al., 2022). *: The protein included in the orthogroup corresponds to a different transcript from the same gene model. †: The protein is the best available match in the version of the annotation used for the orthogroups, but is not identical to the sequence used in the original publication of the protein.

REFERENCES

Kamal, N., Tsardakas Renhuldt, N., Bentzer, J., Gundlach, H., Haberer, G., Juhász, A., et al. (2022). The mosaic oat genome gives insights into a uniquely healthy cereal crop. *Nature* 606, 113–119. doi:10.1038/s41586-022-04732-y

Zhang, P., Zhang, L., Chen, T., Jing, F., Liu, Y., Ma, J., et al. (2022). Genome-wide identification and expression analysis of the GSK gene family in wheat (Triticum aestivum L.). *Molecular Biology Reports* 49, 2899–2913. doi:10.1007/s11033-021-07105-2
